# Arterio-Esophageal Fistula: A Complication of Button Battery Ingestion

**DOI:** 10.7759/cureus.43830

**Published:** 2023-08-20

**Authors:** Raed M Almutairi, Ali I Almania, Saleh Alabood, Mohmmed Alkarzae

**Affiliations:** 1 Otorhinolaryngology, King Fahad Specialist Hospital, Buraydah, SAU; 2 Medicine and Surgery, Qassim University, Buraydah, SAU; 3 Otorhinolaryngology - Head and Neck Surgery, Security Forces Hospital, Riyadh, SAU

**Keywords:** arterio-esophageal fistula, pediatric, hematemesis, foreign body ingestion, button battery ingestion

## Abstract

Button battery (BB) ingestion is one of the rare foreign body ingestion (FBI) emergencies. Nevertheless, it carries high morbidity and mortality rates. In this case, we present a child with button battery ingestion complicated after successful removal by massive hematemesis and cardiopulmonary arrest. The patient was resuscitated and admitted to the intensive care unit (ICU). The event resulted in multiple neurological sequelae as demonstrated by radiological study as well as clinical examination.

## Introduction

Foreign body ingestion (FBI) is a common event in the pediatric group. The most serious form of FBI is button battery (BB) ingestion, which carries high rates of morbidity and mortality [1]. In recent years, the rates of button battery ingestion in children have increased as a result of the variety of household devices that require large batteries that can cause serious life-threatening events [2]. These events or injuries are not limited only to the esophagus but have a wide spectrum of injuries, including damage to adjacent airways, mediastinal structures, and tissues, as well as vascular injuries [1,2]. The severity of button battery ingestion is explained by its different mechanisms, which are electrical injuries, mechanical injuries, and caustic injuries. These mentioned injuries lead to coagulative necrosis that starts as rapidly as 15 minutes of button battery impaction in the esophagus, while major corrosive injury occurs within hours of ingestion [3]. The management of button battery ingestion is the most challenging among all FBI due to the fact that it is associated with complications and a high rate of morbidity and mortality even after BB removal [4]. In our case, we will discuss one of these rare life-threatening complications and compare it with similar case presentations, management, and outcome.

## Case presentation

**Figure 1:**
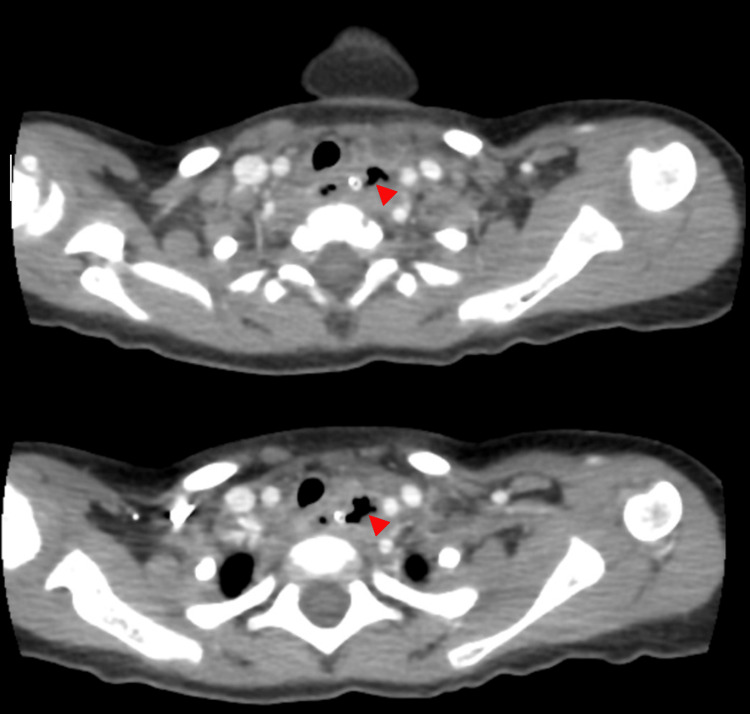
CT of the neck and chest The proximal part of the esophagus, inferior to the thyroid gland, shows an irregular configuration with extraluminal air foci (arrowhead) and rim enhancement, suggestive of focal esophageal perforation. There were no signs of blood vessel injury at the level of the study. CT: computed tomography

**Figure 2:**
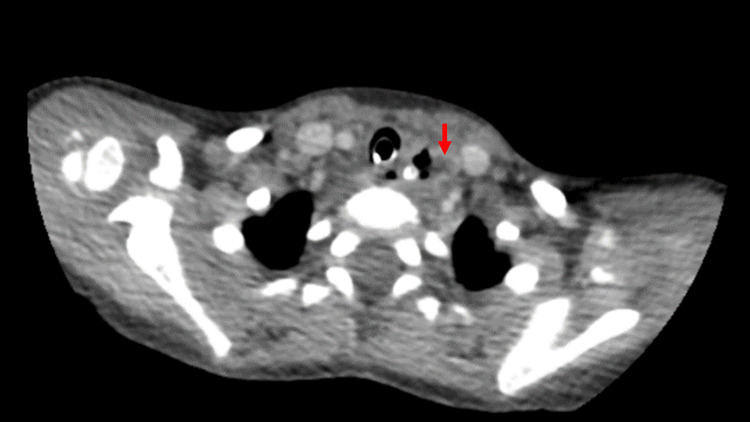
CT of the neck and chest Two days later, newly developed compression of the medial wall of the left common carotid artery caused significant luminal narrowing with intraluminal thrombus (red arrow) at the level of the T1. Here, the carotid-esophageal fistula cannot be totally excluded. The distal aspect of the left common carotid artery and the remaining major vasculature are patent. CT: computed tomography

A previously healthy 20-month-old male presented to the emergency department with a history of greenish vomiting and decreased oral intake for the past 10 days. An urgent chest X-ray was done, and it showed a button battery lodged in the upper third of the esophagus. The patient was vitally stable when presented to the emergency department. The patient was taken immediately to the operating room (OR). Rigid esophagoscopy was done, and the button battery was removed. On examination of the button battery (BB) site, there was dark necrotic tissue at the site of BB impaction in the inner surface of the esophagus, and no active bleeding was noticed. A nasogastric tube (NGT) was inserted and extubated. The patient was stable and was taken to the ward. The following night, the patient developed an episode of hematemesis, which was large in amount. The patient then suffered from cardiac arrest and was coded and resuscitated successfully. Following the event, the patient was intubated after resuscitation, and computed tomography angiography (CTA) was requested. The next day, a contrast CT of the neck and chest was done. Neck and chest CT showed focal esophageal perforation with newly developed compression of the medial wall of the left common carotid artery causing significant luminal narrowing with intraluminal thrombus at the level of T1 vertebra. Carotid-esophageal fistula could not be excluded (Figures 1, 2).

**Figure 3:**
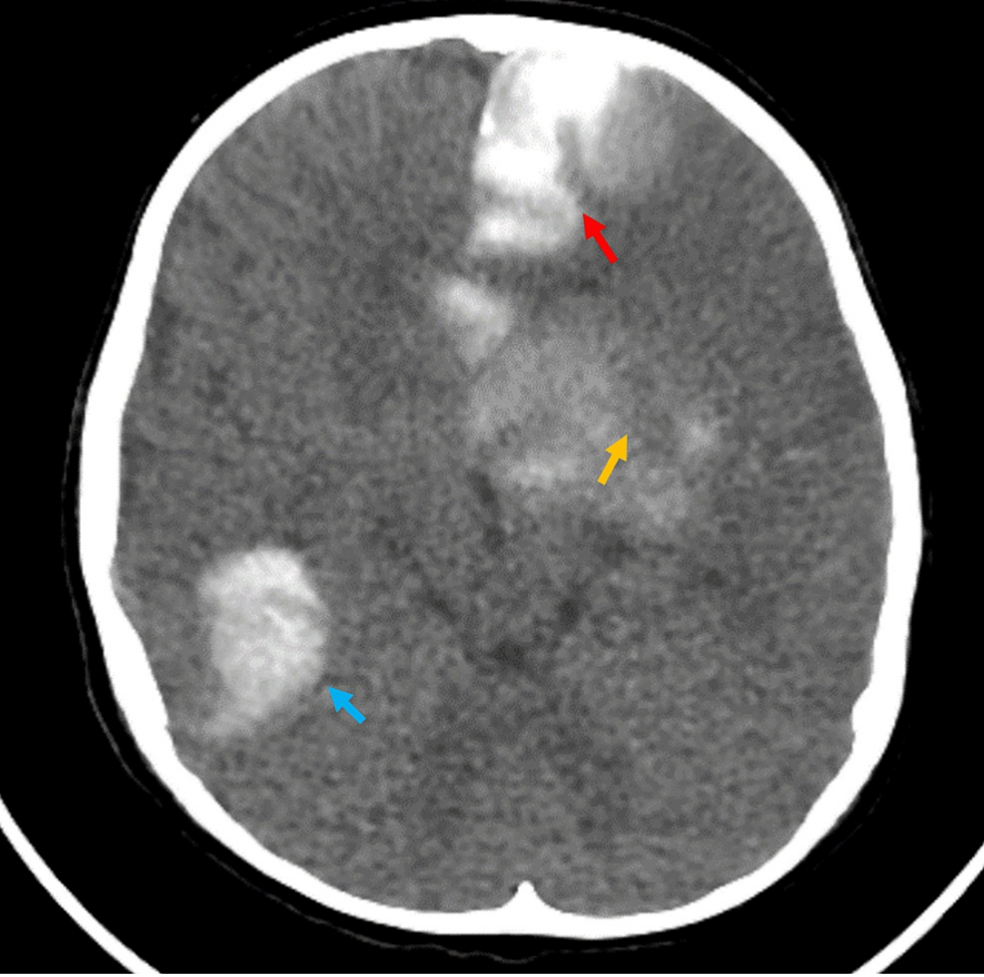
CT of the brain Bilateral intraparenchymal hyperdensities seen in the left frontal extending to the corpus callosum and right temporal lobe (red and blue arrows, respectively) that likely represent contrast extravasation secondary to the previous procedure. Left frontoparietal lobe acute infarction (yellow arrow) with 0.3 cm midline shift to the right side. Blood density in the left basal ganglia that could represent hemorrhagic transformation. CT: computed tomography

Three days later, CT of the brain (Figure 3) showed interval worsening of the left cerebral hemisphere with edematous changes associated with a mild increase of the midline shift, effacement of the left lateral ventricle, and subfalcine herniation with a high probability of uncal herniation.

**Figure 4:**
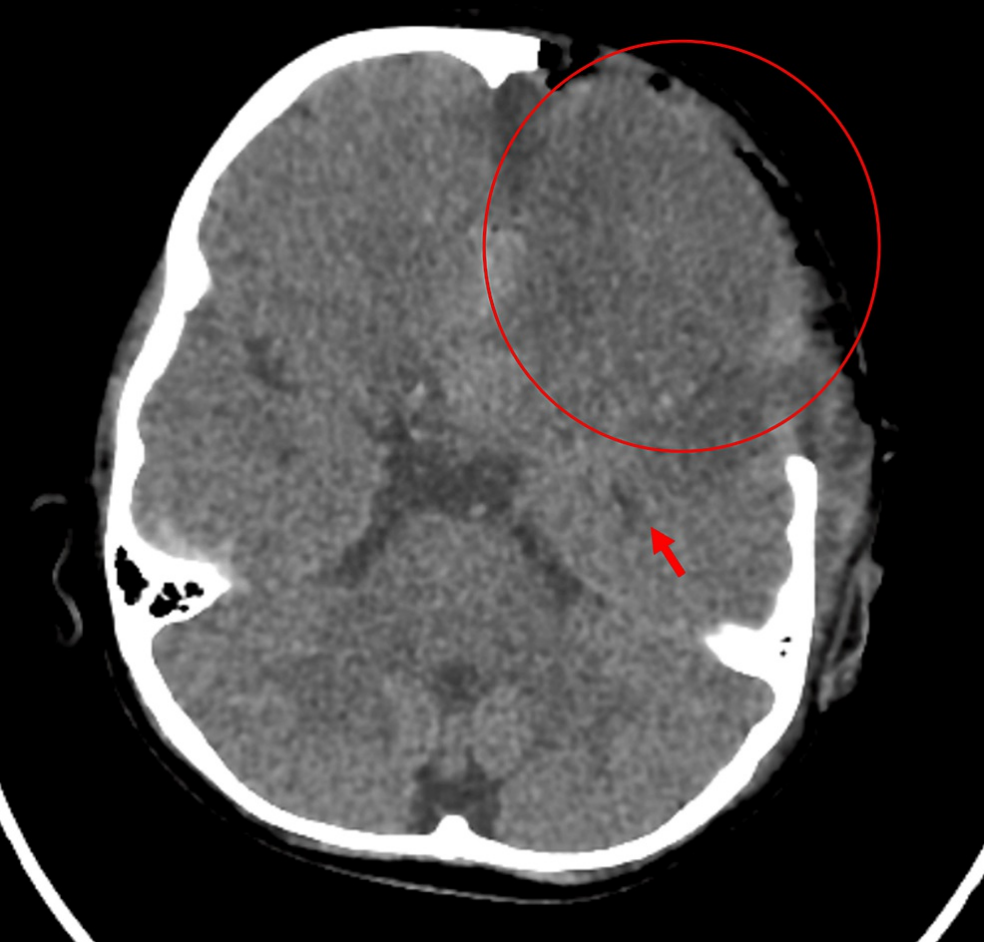
CT of the brain Left hemisphere edematous changes with midline shift S/P left frontoparietal decompression craniectomy (red circle). Effacement of the left lateral ventricle (red arrow). CT: computed tomography, S/P: status post

**Figure 5:**
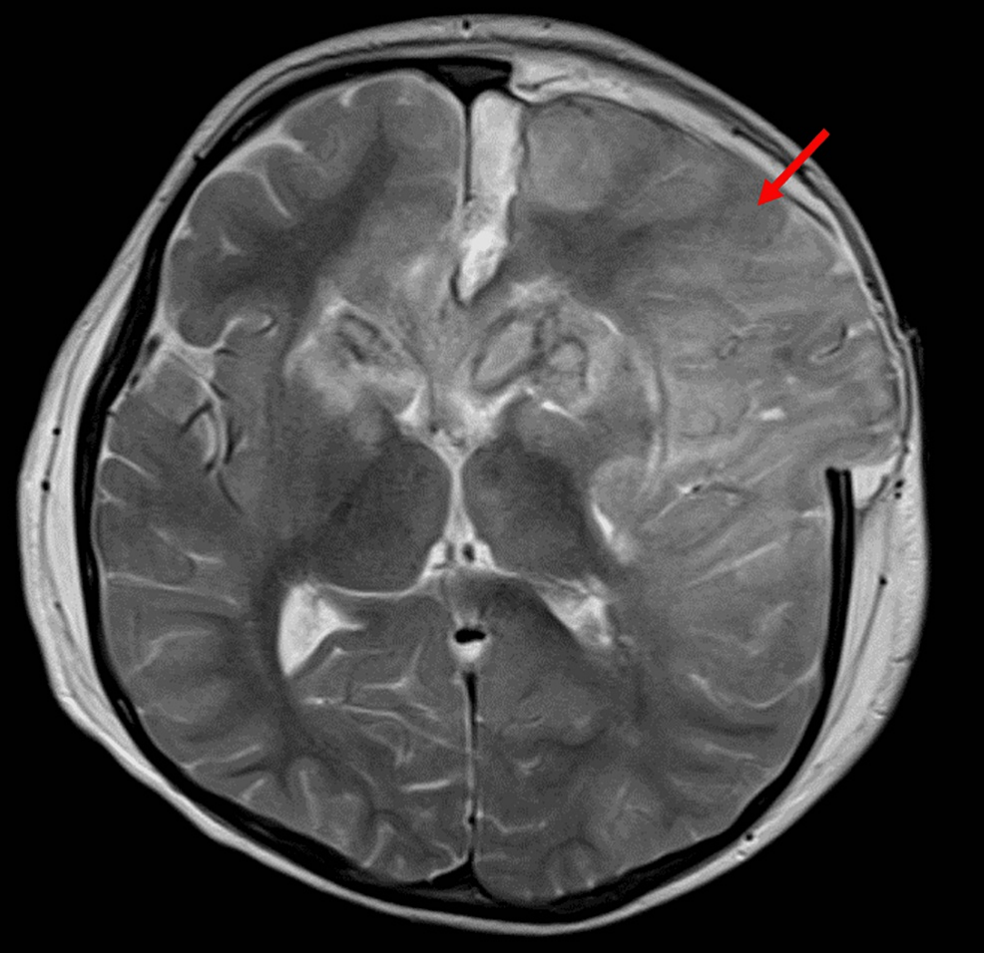
MRI of the brain Left hemisphere edematous changes with midline shift S/P left frontoparietal decompression craniectomy (red arrow). MRI: magnetic resonance imaging, S/P: status post

The patient underwent left frontoparietal decompression craniectomy the next day. CT a day after the procedure (Figure 4) shows interval worsening of the left cerebral hemisphere edematous changes associated with a mild increase of the midline shift and effacement of the left lateral ventricle, as well as subfalcine herniation with a high probability of uncal herniation. There was a significant improvement in the parafalcine hyperdensity and a mild size decrease in the right temporal hyperdensity. Magnetic resonance imaging (MRI) was obtained also a few days after (Figure 5).

**Figure 6:**
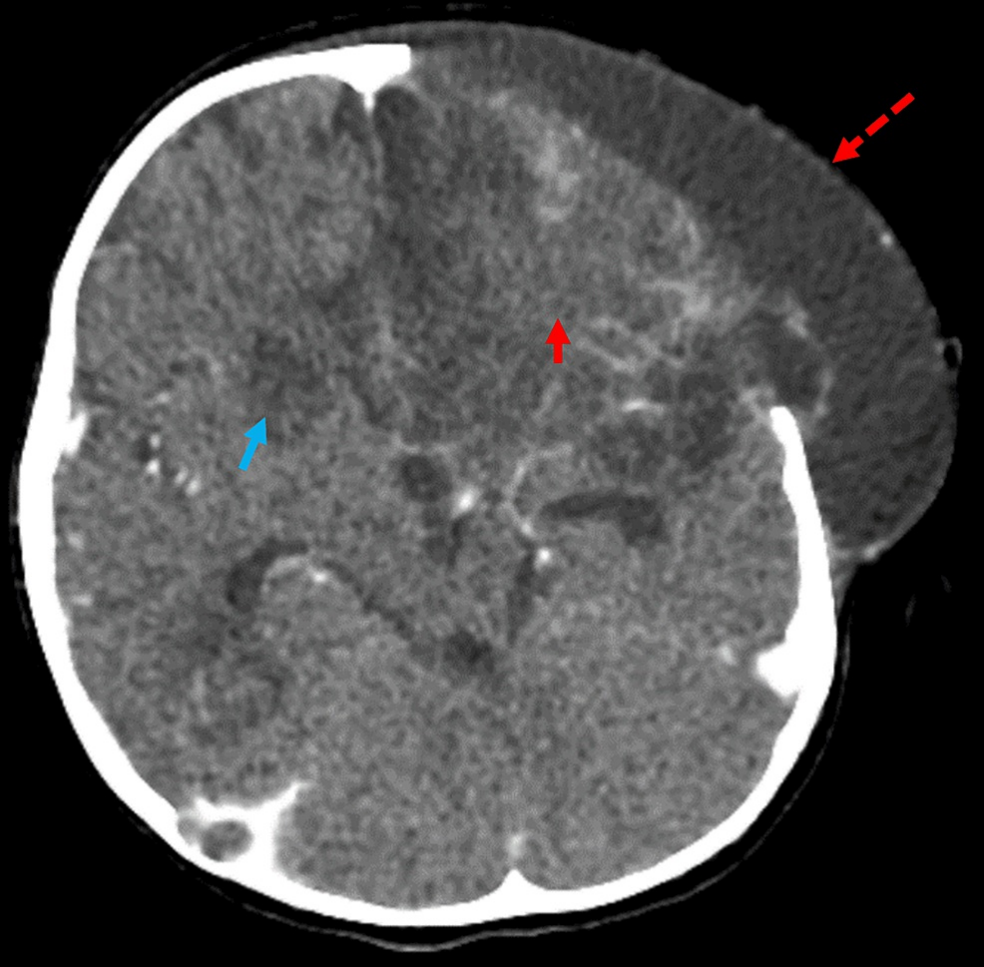
CT of the brain Subgaleal CSF attenuation suggesting hygroma formation (dashed arrow). Left frontoparietal encephalomalacia changes (red arrow). Right frontal encephalomalacia changes (blue arrow). CT: computed tomography, CSF: cerebrospinal fluid

A brain CT 15 days later (Figure 6) demonstrated no evident intra- or extra-enhancing collection formation to suggest abscess formation. There was interval improvement of the left frontal extracranial brain herniation with development of subgaleal cerebrospinal fluid (CSF) attenuation suggesting hygroma formation, as well as progressive increase of the left frontoparietal and right frontal hypoattenuation suggesting encephalomalacia changes.

On follow-up, the patient underwent left and posterior parietal external ventilation drain insertion under general anesthesia, and a month later, the patient underwent left frontal cranioplasty and left ventriculoperitoneal (VP) shunt revision. The patient was discharged home after a month from the last procedure on warfarin and follow-up with hematology and neurosurgery. The patient’s latest recorded visit was three months after discharge, and the patient was well, with left hemiplegia, no seizure, on physiotherapy, and waiting for rehabilitation.

## Discussion

Foreign body ingestion (FBI) in children is a common event. The most frequent objects ingested are coins and bones of fish and chicken, while the prevalence of ingestion of button batteries (BB) is not more than 2% [5]. Complications from FBI of BB have a broad spectrum, including pneumonia, mediastinitis, hemorrhage, esophageal ulceration, sepsis, tracheoesophageal fistula, perforation of the esophagus, pneumoperitoneum, tension hydropneumothorax, and esophageal-vascular fistulas, such as erosions into thyroid vessels, subclavian artery, arterio-esophageal fistula (AEF), and esophageal stenosis [4,6]. The presentation of these complications can be as early as 10 hours of button battery ingestion or present late at 28 days, with most patients presenting on day 9 or 10 [6]. One of the rare serious complications of button battery ingestion in the esophagus is arterio-esophageal fistula (AEF), especially if the BB was impacted in the mid-esophagus. However, the National Capital Poison Center (NCPC) database reported that AEF can occur at all esophageal levels, including fistula with the aorta, carotid, subclavian, and thyroid arteries. According to the NCPC data report taken from a total of 71 deaths in children after BB ingestion worldwide since 1977, the most common underlying etiology was as a result of arterio-esophageal fistula [6]. According to previous data, serious complications are more common in unwitnessed events, children who are younger than six years old, and batteries size bigger than 20 mm [7].

This case highlights several key points in regard to clinical management, rare complications as a result of BB ingestion, late presentation, and the importance of a multidisciplinary team approach, finally comparing it with other similar complications. There are many important factors other than the location of BB impaction in the esophagus that leads to AEF, including time length prior to BB removal and negative battery pole orientation [8]. Current guidelines from the North American Society of Pediatric Gastroenterology, Hepatology, and Nutrition (NASPGHAN) stress on the removal of the esophageal button battery within two hours of ingestion [9]. In our case, the length of time prior to BB removal was delayed significantly up to approximately 10 days, and the child’s age is less than six years old. Both factors are considered important for the development of AEF. A new intervention of administering honey or sucralfate at the time of recognition can be given to a similar case to decrease the degree of mucosal injury [10], but due to the sparsity in the literature on the benefits in changing the outcome, in relation to our case, specifically, it was not performed, although we believe that this type of intervention is important for the general population to know how to manage in a household environment as it can be a helpful factor in preventing the progression of tissue destruction. The fistula in our case was not clearly seen with CTA, but the incident of hematemesis and the CTA findings both were significant to raise the suspicion of AEF, which guided us to contact a neuro-interventional radiologist for immediate intervention.

## Conclusions

AEF is a rare and life-threatening complication of button battery ingestion with a high mortality rate. The diagnosis of such cases requires a high index of suspicion, clinical expertise, and knowledge of the natural course of this type of case. However, in our case, there was a complication that we did not expect and could not find in the literature. Importantly, this case demonstrated the positive outcome of multidisciplinary efforts in recognizing and managing the serious complications resulting from BB ingestion.
